# The Association Between Childhood Experience of Living with a Heavy Drinker and Self-Rated Mental Health in the Adult General Population

**DOI:** 10.3390/diseases13020028

**Published:** 2025-01-23

**Authors:** Danica Romac, Varja Gaić Đogaš, Ljiljana Muslić, Sandro Krašić, Marija Kušan Jukić, Sanja Musić Milanović

**Affiliations:** 1 Teaching Institute of Public Health “Dr. Andrija Štampar”, Mirogojska 16, 10000 Zagreb, Croatia; danica.romac@stampar.hr (D.R.); marija.jukic@stampar.hr (M.K.J.); 2 School of Medicine, University of Split, Šoltanska 2a, 21000 Split, Croatia; 3 Croatian Institute of Public Health, Rockfellerova 7, 10000 Zagreb, Croatia; ljiljana.muslic@hzjz.hr (L.M.); sanja.music@hzjz.hr (S.M.M.); 4 Faculty of Humanities and Social Sciences, University of Zagreb, Ivana Lučića 3, 10000 Zagreb, Croatia; sandro.krasic.sk@gmail.com; 5 School of Public Health “Andrija Štampar”, School of Medicine, University of Zagreb, Rockfellerova 7, 10000 Zagreb, Croatia

**Keywords:** alcohol drinking, childhood experience, mental health, preventive medicine

## Abstract

Background and Objectives: Considering the link between childhood experiences with adult health and well-being, this study examined how living with a heavy drinker (HD) during childhood affected self-rated mental health (SRMH) in adulthood, while identifying risk and protective factors and assessing the prevalence within a regional context. Materials and Methods: Data (N = 11,113) were obtained from a cross-sectional DEEP SEAS survey (2021) of the general population, aged 18–64 years, in six countries (Croatia, Bosnia and Herzegovina, Slovenia, Austria, Hungary, and Italy). Results: A statistically significant difference in SRMH was found, related to the childhood experience of living with an HD (MD = −0.221, 95% CI −0.250–0.172, N = 10,886) and being negatively affected (MD = −0.216, 95% CI −0.311–0.122, N = 2978). The correlation analysis revealed that individuals who lived with an HD during childhood perceived poorer SRMH in adulthood, consistently across all observed countries. Subsequent logistic regression identified different predictors for SRMH between those who were negatively affected and those who were not. The relationship satisfaction (RAS) was the strongest predictor, significantly contributing to better SRMH, especially in the subgroup that was not negatively affected (OR 28.724, 95% CI 3.450–239.173). A high prevalence of individuals negatively affected was found, especially in Hungary (34.4%) and Croatia (26.5%). Conclusions: Growing up with someone who was a heavy drinker may have lasting negative consequences on SRMH, with a negative subjective evaluation of mental health in adulthood. Targeted public health and preventive measures are needed to protect those living with heavy drinkers.

## 1. Introduction

Alcohol consumption is an important risk factor contributing to the global burden of disease. Its effects extend beyond individuals who consume alcohol excessively, by negatively affecting those in the drinker’s household and social environment, so people who have a heavy drinker in their lives experience reduced subjective mental health [1,2,3,4].

Young people are especially vulnerable to the effects of others’ drinking. There is a large body of evidence showing that children who grew up in families with parental alcohol abuse are at a higher risk of developing a wide range of psychological and physical health consequences in the short and long run. They also tend to fare poorly in important areas of life, such as education and social relationships [1,3,5,6]. There is a consensus amongst researchers, clinicians, and policymakers that parental alcohol abuse negatively affects child well-being [7]. Previous research also indicates that parental subclinical drinking tends to be positively associated with drinking and related harm in offspring [3].

Although it has been less studied, alcohol use by other household members may similarly harm children, which may track into adulthood. Children who grew up in homes with alcohol issues have been shown to be prone to later adverse effects in several areas in life, including substance abuse, behavioral problems, and poor physical and mental health [8]. Additionally, excessive alcohol or substance use by relatives can harm children mentally, physically, and socially, increasing their risk of alcohol and other substance use in their adult years [2]. Globally, harm caused by family members’ alcohol abuse is associated with poor mental health and reduced quality of life for others in the family [9].

It is well-known that children in families with alcohol issues experience more frequent negative life events (re)occurring during childhood, commonly referred to as adverse childhood experiences (ACEs). These are conceptualized as child maltreatment and cover a variety of (re)occurring events before the age of 18, including emotional and/or psychical abuse or neglect, or growing up in a home where one or both parents were affected by substance use. Individuals with a history of ACEs tend to have significantly more life-course health problems, with long-lasting effects on health being known to contribute to premature morbidity and mortality [6,10,11,12,13]. Review studies and meta-analyses have shown that exposure to ACEs is directly and inter-generationally associated with poor mental and physical health outcomes, resulting in considerable economic costs to society [14,15]. Critically, more than one in five adults across Europe and North America report suffering at least one ACE during childhood. Given such high proportions, it is crucial to understand how ACEs may affect health and behavior [11].

Despite the evidence of the negative mental health consequences associated with alcohol use within the home, several gaps about alcohol’s harms from various other sources, and their effects on mental health, are under-researched. There is a large knowledge gap in the understanding of the nature and mechanisms through which others’ drinking negatively affects someone else, as well as the pathways for access to help. Most studies primarily focus on short-term consequences, such as offspring alcohol use, heavy episodic drinking (HED), or intoxication, while long-term consequences and underlying mechanisms remain largely unexplored.

However, none of the previous studies comprehensively investigated the different dimensions of childhood and/or teen exposure to heavy drinkers and their relationship with self-rated mental health (SRMH) in the adult general population.

To address the gaps in alcohol’s second-hand harms, this study investigated how patterns of childhood exposure to others’ drinking in the home environment were related to SRMH over time. This knowledge is crucial for prevention and intervention strategies to reduce alcohol-related harms, both among general groups, and especially vulnerable groups, like children and teens affected by others’ drinking. Identifying underlying factors that increase the risk of mental health problems is key to improving prevention, early intervention, and treatment strategies [6].

Because many mental health conditions remain undiagnosed, self-rated assessment tools may capture a more comprehensive understanding of mental health in the general population and help identify those in need of mental health resources and at risk of alcohol-related harm. SRMH is a strong predictor of health outcomes and well-being, independent of objective measures, validated in adults as a useful and reliable indicator for monitoring the general mental health of the population that can reflect one’s overall mental well-being [16]. As the single-item measure of SRMH is strongly associated with common measures of mental health conditions, it can be useful for general health screening surveys, but also to facilitate the early recognition of population-wide mental well-being [17]. Moreover, general population surveys provide the prevalence and distribution of alcohol-related harm among those experiencing both subclinical and clinical parental drinking during childhood and/or adolescence.

Thus, using a large, nationally representative sample that represents the drinking cultures of six countries in the areas of southeast and central Europe, the current study explores SRMH in relation to growing up with a heavy drinker (HD). Focusing on a broader context could reveal which factors may be risky or potentially protect SRMH.

The study aimed to develop an understanding of alcohol-related harms resulting from others’ drinking on SRMH, through the following main objectives:

1. To examine the associations between the experience of living with an HD during childhood and/or teenage years and the risk of poor SRMH in participants’ adult years, as well as to analyze differences between those who lived with an HD and those who did not, focusing on socioeconomic, drinking behaviors, and health well-being predictors.
2. To analyze the differences between two exposed subgroups—those negatively affected and those not negatively affected—adjusting for health well-being, drinking behaviors, and socioeconomic (SES) predictors, to identify risk and protective factors for SRMH.

Additionally, we aimed to provide insights into the prevalence to address the broader scope of these issues and to explore the magnitude and cross-country differences through the following secondary objective:

- To determine the prevalence of participants who lived with an HD during their childhood and/or teenage years and to assess how many of them were negatively affected within a regional context across six countries in southeast and central Europe.

The paper assumed that exposure to HD or someone who drank a lot during childhood/teenage years within the home environment may affect long-term SRMH, guided by evidence indicating a link to poor mental health [18,19]. It was expected that the relationship between the childhood experience of living with an HD and subjective mental health in adulthood would differ between subgroups exposed to an HD based on specific characteristics. The study proceeded with the following hypotheses (H1 and H2):

**H1.**  *Living with an HD or someone who drank a lot during childhood and/or teenage years negatively affects self-perceived mental health in adulthood, leading to higher rates of poor SRMH compared to those who did not live in a home drinking environment, both in the overall sample and in each country individually. There are also differences in drinking behaviors and health well-being predictors between the two groups.*

**H2.**  *There is a difference between individuals who experienced living with an HD and were negatively affected, and those who reported that they were not negatively affected regarding social and health-behavioral predictors, which are assumed to play an important role in subjective mental health.*

## 2. Materials and Methods

### 2.1. Data

Data were obtained from the cross-sectional DEEP SEAS survey, designed to represent the general adult population (18–64 years), from 33 European countries, of which data from 6 countries were used in this study. The research was conducted on 11,113 participants (from Austria—3095; Bosnia and Herzegovina—1500; Croatia—1502; Hungary—2009; Italy—1504; and Slovenia—1503 participants). This was the initial sample size, and for all subsequent analyses, respondents with missing data were excluded pairwise from the study. The final number of respondents for each study and the characteristics of the sample, categorized by country, can be found in Table 1.

The results from this group of countries, located in southeast and central Europe, all geographically close and sharing centuries-long history, were analyzed to provide insights into regional trends, drinking behaviors, and social dynamics. Their diversity enhances the research strength and scope by offering more nuanced and detailed data for a better understanding of lasting second-hand alcohol-related harms within the regional context.

The survey was deployed from January to March 2021, with a high level of harmonization across countries regarding the content of the standardized SEAS-2 survey questionnaire and quality controls. Quota sampling was performed by the market research company Kantar [20], with participants’ distribution in the specific country matching the target population by gender and age. Some groups were excluded from sampling frames, like the homeless or people living in an institution.

The mode of administration of the survey was Computer-Assisted Web Interviewing (CAWI), except in Bosnia and Herzegovina (BIH), where it was conducted by Computer-Assisted Telephone Interviewing (CATI). The final versions of the questionnaire in every language were revised twice by national experts before the survey was launched.

At the beginning of the study, participants were clearly informed about the research and its purpose. They were provided with a detailed explanation to ensure they understood the nature of the study and the questionnaire before giving their consent.

The owner of the SEAS-2 data and translated questionnaires is the Health and Digital Executive Agency (HaDEA) of the European Commission. The DEEP SEAS group, as contractors, are authorized to share these data and supporting files publicly, in line with the HaDEA’s policy to provide open access to documents and data created with EU funding. The DEEP SEAS questionnaire is available online at https://www.deep-seas.eu/standard-eu-alcohol-survey/, accessed on 16 May 2023.

### 2.2. Measures

#### 2.2.1. Outcome Variable

Self-rated mental health (SRMH), as a dependent variable, was assessed using a single-item measure. Respondents were asked to rate their mental health, as a component of well-being (WB), on a five-point scale, with the response categories ranging from very good, good, fair, and poor to very poor. The present study’s response categories were dichotomized as 0 = good SRMH (good and very good) and 1 = poor to fair SRMH (very poor, poor, fair).

#### 2.2.2. Exposure Variables—Main Predictors and Other Explanatory Variables

Independent variables were divided into the following categories: sociodemographic, health and well-being variables, and variables concerning alcohol.

Several sociodemographic variables were included in the study as covariates, based on the published literature [2,18].

Gender was categorized as the following two groups: (1) males and (2) females, given the small number of respondents indicating other gender (n = 14).

Age was categorized as the following three groups: (1) 18–34; (2) 35–49; and (3) 50–64 years.

Level of education was categorized as (1) low (completed primary school or less); (2) medium (completed secondary school); and (3) high education (at least a short study at university or more).

Professional activity was assessed by asking respondents if they are professionally active or non-active, as follows: (1) non-active; (2) active.

People living in household was measured based on how many persons they lived with, categorized as follows: (1) living alone; (2) living with at least one person. Those who lived alone were compared to those that lived with at least one household member.

Living with a heavy drinker (HD) or a person who drank a lot in the household during childhood or teen years served as the control variable of interest, allowing us to divide the survey sample and perform statistical analysis. The respondents were asked if they, as a child or teenager, lived with someone whom they considered to be a fairly heavy drinker or someone who drank a lot (e.g., father, mother, their partners, siblings, or other household members) (CH_1). Then, those who reported living with a fairly heavy drinker during childhood and/or adolescence were asked how much they were negatively affected by this or these persons’ drinking (a lot/a little or not affected at all) (CH_2). The sample was then divided into the following two groups: those who did not live with a heavy drinker during their childhood and teen years, and those who did. Additionally, the group that lived with a heavy drinker was further divided into the following two groups: those who were negatively affected and those who reported not being negatively affected. Originally, this variable had three answer options, which were as follows: “Were you affected a lot, a little or no affected at all”, which were dichotomized as 1 = not affected and 2 = affected, so “affected a lot” and a “little affected” formed the category “affected”.

To assess participants’ own alcohol drinking behaviors for the past 12 months, the following two indicators were used from the DEEP SEAS main study questionnaire: frequency of drinking and heavy episodic drinking (HED).

The overall frequency of alcohol consumption (F1) offered data about usual overall consumption and about abstainers. Respondents were asked to indicate how often they drank any beverage containing alcohol, including beer, wine, or spirits, even in small amounts, in the past 12 months. The frequency of alcohol use was analyzed with the following categories: (1) daily or almost daily; (2) at least once a week (weekly); (3) at least once a month, but less often than once a week (monthly); (4) less than once a month (less frequently); and (5) never in the past 12 months (abstainers).

Heavy episodic drinking (HED) was assessed by reporting risky single occasion drinking (RSOD) in the past 12 months. Participants were categorized as (1) abstainers, (2) non-risky drinking, or (3) 1+ time(s). Heavy episodic drinking (HED) was defined as reporting at least one episode of drinking more than 40 g (women) or 60 g (men) of pure alcohol on a single drinking occasion during the past year. This was assessed in gender-specific questions, such as the following: ”How often in the past 12 months, have you had 40 g (for women) and 60 g (for men) of pure alcohol or more than one occasion?” The country-specific number of drinks has been entered, which corresponds to 40 g of 100% alcohol for women (about 2.5 pints of regular beer or lager, 4 small glasses of wine, 4 single measures of spirits, or combinations of different beverages) and 60 g of pure alcohol for men (about 4 pints of regular beer or lager, 3 medium glasses of wine, 6 single measures of spirits, or combinations).

We also included the variable of personal drinking consequences, also known as individual harms for a drinker (RAPS). A short, four-item screening instrument was studied, called the ‘Rapid Alcohol Problems Screening Test’ (RAPS), which can identify problematic drinking. The RAPS scale consists of four simple questions dealing with unwelcome consequences of drinking in the past 12 months, with a total score ranging between 0 and 4 and including the following: feeling guilty, blacking out, failing to do what was normally expected, and early morning alcohol consumption. Concerning personal drinking consequences, participants ranged from 0 to 4, with a greater number reflecting greater harm (RAPS).

Health well-being variables used in the study, such as self-rated health (SRH) and relationship with others (RAS-1), were measured using a single-item measure. Respondents were asked to rate their health (“How is your health in general?”) and relationship satisfaction (“How would you generally rate your satisfaction with your relationships with people around you i.e., your family, friends, and colleagues?”) on a five-point scale, from very good, good, fair, poor, to very poor; for the present study, these were dichotomized as 0 = good (good and very good) and 1 = poor to fair (very poor, poor, fair).

### 2.3. Statistical Analyses

Statistical analysis was carried out using SPSS Statistics ver.23.0 (ID: 729038; IBM Corporation, Chicago, IL, USA).

Initially, a descriptive analysis was conducted to observe the characteristics of the total sample, as well as the specific subsamples of participants from different countries—Croatia, Bosnia and Herzegovina, Slovenia, Austria, Italy, and Hungary—including an overview of sociodemographic variables, drinking behaviors, and health-related factors across all observed countries. The analysis specifically focused on those who reported living with a heavy drinker (HD) compared to those who did not, to identify statistically significant differences and associations between the experience of living with an HD and self-rated mental health (SRMH) in adulthood, with an emphasis on prevalence.

Secondly, a *t*-test was used to examine the difference in self-rated mental health (SRMH) outcomes between participants who experienced living with a heavy drinker and those who did not. For those who experienced living with a heavy drinker, a *t*-test was calculated to determine if a difference in self-perceived mental health existed between those who reported that they were negatively affected and those who did not.

Furthermore, we proceeded to build the logistic regression model. We calculated Spearman’s correlation to identify which variables are statistically significantly associated with self-rated mental health (SRMH) for four subsamples. The total sample was divided into two subsamples by variables concerning experience with a heavy drinker during childhood/teenage years (whether or not the participants experienced living with a heavy drinker during childhood/teen years). We explored the differences between these groups in more detail, focusing on how other factors relate to SRMH in adulthood to identify risk and protective predictors of SRMH. Specifically, we examined which factors are important in explaining why certain groups (those who did or did not live with an HD) perceive their mental health in adulthood as worse.

Finally, a subsequent additional analysis was conducted on two subgroups of those who experienced living with heavy drinkers and reported they were or were not negatively affected by them. Additionally, we analyzed which factors are significant for SRMH in adulthood among those who lived with an HD, comparing those who did experience negative effects from living with an HD to those who did not, looking for protective and risk factors.

Conclusively, we conducted four logistic regression analyses, separately, with SRMH as the dependent variable and all independent variables entered simultaneously. Variables that were found to be statistically significant within the Spearman correlation analysis were included in logistic regression models as independent variables.

The odds ratio (OR) and 95% confidence intervals (CI) were calculated to explore associations, while Nagelkerke R square was calculated to explore goodness of fit. Statistical significance was analyzed at *p* < 0.05.

## 3. Results

### 3.1. Descriptive Analysis for the Overall Sample

The distribution of the overall sample, as well as country-specific data which refer to sociodemographic, alcohol-related, and health well-being variables, is presented in Table 1.

The prevalence of living with an HD, or someone who drank a lot sometimes, during childhood/teen years varied across the six countries, with the highest rates in Hungary, then in Croatia, and the lowest in Italy. Most respondents reported being negatively affected, across all countries. Respondents from Hungary (34.4%) most often experienced living with a heavy drinker during childhood with negative effects, and they also reported the worst SRMH among all observed counties (Table 1).

Among the surveyed countries, respondents from Croatia exhibited the highest prevalence of risky drinking behavior, with 76.7% reporting heavy episodic drinking (HED) in the past 12 months and one or more adverse personal consequence attributed to alcohol consumption, according to the RAPS scale (32.7%).

### 3.2. Preliminary Analysis

#### 3.2.1. Testing the Statistical Significance of Differences in Self-Rated Mental Health (SRMH)

Results from Student’s *t*-test (Table 2) suggest that people who experienced living with a heavy drinker (HD) in their childhood and/or teenage years had poorer SRMH than those who did not. Furthermore, we found a statistically significant difference with respect to SRMH for those who were negatively affected by HD compared to those who were not negatively affected. Those who were negatively affected had poorer SRMH.

#### 3.2.2. Test for Association with SRMH

In the correlation analysis, we found a statistically significant correlation between the experience of living with a heavy drinker during childhood and/or teenage years (CH1) and SRMH, across all countries of interest (r = 0.100; *p* < 0.01), (Table 3). This correlation suggests that individuals who experienced living with a heavy drinker during childhood tended to perceive poorer SRMH in adulthood, a pattern consistent across all countries.

When we assessed only those groups who experienced living with a heavy drinker, we found that if an individual had been negatively affected by this experience, the self-assessment of SRMH tended to be poorer in their adult years, compared to those who were not negatively affected (r = 0.080; *p* < 0.01). While we observed a significant correlation in the overall sample (N = 10,886), we did not observe a significant correlation across all countries.

### 3.3. Correlates of SRMH

Furthermore, four logistic regression analyses were conducted. Specifically, an additional analysis of the relationship with SRMH was performed for two samples based on exposure to heavy drinkers, distinguishing between those who lived with a heavy drinker and those who did not (Table 4). Two analyses were conducted on HD-exposed groups, regarding the influence of HD (Table 5), including variables previously shown to be related to SRMH (Appendix A).

#### 3.3.1. Results of Logistic Regression Analysis According to the Experience of Living with a Heavy Drinker (HD) During Childhood and/or Teen Years

The logistic regression results (Table 4), regarding the experience of not living with a heavy drinker in childhood, indicated that SRMH is better in males, older individuals, and those who are professionally active (i.e., have a job). These participants generally rate their own health (SRH) and relationships with others (RAS-1) more positively, had lower heavy episodic drinking (HED) in the past 12 months, non-risky drinking, and experienced fewer individual harms from drinking (RAPS). Additionally, more frequent drinking is linked to worse self-rated mental health (compared to those who drink rarely).

For those who experienced living with a heavy drinker in childhood, better self-rated mental health (SRMH) was found in males, older individuals, those who had a job, those who reported better general health (SRH) and relationships with others (RAS-1), and those who had fewer individual harms from drinking (RAPS). Again, relationships with others (RAS) and self-rated general health (SRH) were the best predictors, followed by gender. Women who experienced living with a heavy drinker in childhood/teen years had a 1.5 times higher chance of poorer SRMH in adulthood than men. The drinking habits, such as frequency of alcohol drinking and HED, did not show a statistically significant correlation in this group. The RAPS questionnaire, which assesses personal alcohol consequences, indicated better mental health outcomes with lower scores.

#### 3.3.2. Results of the Logistic Regression Analysis According to the Influence of a Heavy Drinker During Childhood and/or Teen Years

The additional regression analysis, which used subgroups based on different perceptions of HD influence, provided additional insights into the relationship between each indicator and self-rated mental health (SRMH), helping to identify potential protective and risk factors for SRMH and differences between subgroups.

The results for those who experienced living with a heavy drinker during childhood and reported that they were not negatively affected indicated that SRMH is better in those who rated their own health in general (SRH) and relationships with others (RAS-1) more positively, and had no risky drinking habits, such as HED, in the past 12 months (Table 5).

For individuals who experienced living with a heavy drinker in childhood and reported that they were negatively affected, better self-rated mental health (SRMH) was found in males, those having a job, those who reported better general health (SRH) and relationships satisfaction (RAS) with others, those who had lower heavy episodic drinking (HED) in the past 12 months, and those who had fewer individual harms from drinking. Those respondents who reported that they were negatively affected and were professionally active had a 41% higher chance of better SRMH than those who were not professionally active.

Age group and frequency of drinking were not identified as significant predictors in the exposed individuals, and education level dropped out as well in this context of variables.

## 4. Discussion

The results from this study revealed that the experience of living with a heavy drinker (HD), or someone who drank a lot, during childhood and/or teen years was significantly associated with an increased risk of poor self-rated mental health (SRMH) in adult years. In other words, growing up with relatives such as parents, step-parents, or siblings who drank a lot within the household may have negative long-term consequences on SRMH, and implies that there are not only lasting negative effects, but also a potential link between the household drinking environment during childhood and negative subjective evaluation of one’s mental health in adulthood. Due to SRMH’s ability to predict future health outcomes and well-being, the results could indicate that people who were exposed to HD during childhood/teen years could potentially be at a higher risk of developing future mental health problems. These findings were expected and align with previous studies demonstrating that long-term effects and risks of adverse health outcomes in adulthood can occur in individuals living in difficult circumstances, such as being brought up with a relative excessively using alcohol [1,2,3,13].

Existing evidence suggests that heavy drinking can lead to inadequate parenting, increasing the risk of traumatic experiences, such as child abuse or neglect, which may result in negative health outcomes [1,3,21,22]. The literature indicates that the stress of living with a parent or relative who excessively uses alcohol is associated with both short- and long-term harm, elevating the risk of psychological and psychiatric issues. This can have lasting negative effects on health, well-being, education, and job potential [1,2,6].

The pathways from parental alcohol use and adverse health outcomes may involve more complex mechanisms, such as the living environment, the upbringing they receive, and hereditary factors [1,2,12,23]. Understanding these mechanisms is crucial for addressing the multifaceted impact of excessive alcohol use within families.

However, evidence shows that alcohol consumption, even in the absence of dependence or abuse, may change how parents behave around their children. It can cause changes in mood, impaired cognition, impulsivity, and aggression, all of which may contribute to poorer parenting. For example, parents under the influence of alcohol may become less attentive to their children’s needs and misinterpret situations, which may lead to verbal conflicts and/or physical harm to the child [8]. Children can experience psychological violence through negative comments, lack of encouragement or support, indifference, and minimal intimacy [3,8]. The literature also suggests that adults with more exposure to adverse childhood experiences (ACEs) are at a greater risk for poor mental health [18,19,24]. Therefore, preventing childhood maltreatment and addressing wider psychiatric risk factors in individuals exposed to maltreatment could help prevent psychopathology [14,25].

According to the results, the significant negative association between exposed participants (CH1) and SRMH is maintained across all observed countries, with consistent negative effects on SRMH (Table 3). This enhances the validity and generalizability of the results, offering valuable insights for mental health interventions.

Previous studies noted that the strength of the relationship could potentially decrease over time, as there are increasing opportunities for other cognitive, biological, and environmental variables to exert an effect [5]. This knowledge could partially explain the weaker connection in this study (r = 0.100 *p* < 0.001). Although small in size, the effect of an HD during ones’ childhood/teen years on subjective adult mental health among all of the observed countries was consistently significant and has a clear public health message.

The results showed that the childhood experience of living with relatives who excessively use alcohol did not have the same effect on everyone in their adult years. The correlation estimates revealed the following two distinct groups, with different patterns of experience: those who were negatively affected by HD and those who were not.

The observed exposed subgroup of those who were negatively affected constituted a larger proportion across all countries and tended to report worse SRMH in total (CH2), in comparison to those who were not negatively affected (r = 0.080 *p* < 0.001). However, we did not observe a significant correlation in all of the countries studied, which may suggest cultural differences (Table 3). Location, a probable proxy for sociocultural realities, had a statistically significant difference on the relationship with SRMH for exposed subgroups among observed countries (CH2). However, to truly dissect geographic and sociocultural patterns would call for a deeper exploration of each country’s norms than this study permits. Although results for the exposed group (CH2), in total, suggested an association with poor mental health outcomes, further cross-cultural research is needed to clarify the consistency and strength of this relationship, as well as differences between those who were negatively affected and those who were not. Future studies should explore individuals who report no negative effects from others’ drinking and their mental health to better understand resilience, defense, or perhaps denial mechanisms in the field of mental health and alcohol-related problems.

To strengthen correlational understanding, we adjusted for a number of covariates and analyzed variations in prediction between subsamples, assessing the role of other risk and protective predictors (Table 4). In the further regression analysis, we identified that self-perceived relationship satisfaction (RAS) and self-rated general health (SRH) were highly positively associated with SRMH as important predictors that contribute to better SRMH, which is in line with previous studies [26,27].

The most striking connection was between RAS and SRMH, especially among those who were not negatively affected by HD, representing a significant protective factor that could potentially reduce risks for poor SRMH. The persistent statistical significance of RAS, included in the regression model, indicated its important contribution to SRMH and a unique, meaningful association. Accounting for other variables, RAS could be an indicator that is more related to mental health than other variables, improving one’s current SRMH status and probably having the potential to buffer the negative effects on SRMH. These results are in line with others that have documented the link between high relationship satisfaction, and greater emotional and psychological well-being [26]. Prior research has also shown that meaningful and supportive personal relationships have tremendous value, and that social support systems may mitigate the negative effects of ACEs for adult health and well-being [10].

It was unexpected that the variable ‘people living in household’ was not statistically significant in the regression analysis and context of these predictors, for those who did not live with an HD, indicating that the quality of relationships and satisfaction with them were more important than having a family member present in the household.

From a prevention and treatment perspective, it is essential to identify factors that can buffer the link between adverse childhood experiences and poor mental health resulting from HD exposure. Social support, which helps manage stress through psychological and material resources, is known to benefit mental and physical health, and can mitigate the impact of trauma and adversity. Promoting protective factors that build resilience may reduce the impact of less adaptable social vulnerabilities [28]. The observed lasting effects suggest that fostering social support could benefit interventions targeting adolescents and adults. Still, there is a complex interaction between vulnerability and protective personal and social resources that, at times, can mitigate the harm of adversity [10,12,23,28]. It is necessary to note that our indicators did not measure the trauma involved in the experience of living with an HD, such as emotional and/or physical neglect or abuse, often included in ACEs [23].

Recognizing that socioeconomic status (SES) is an important determinant of health [28], we explored the differences in SES among groups that may contribute to their respective SRMH. Professional status, as an alternative SES indicator, was positively associated with SRMH, as expected (Table 4). Being professionally active predicted better SRMH, regardless of HD exposure; still, in the sensitivity analysis, it was significant only for those who were negatively affected (Table 5). It is recognized in the literature that social policies which increase social welfare, access to affordable education, and job opportunities can mitigate collective exposure to childhood adversity [12].

Importantly, we found that education was a significant predictor of SRMH, only in the group of those not negatively affected, but became insignificant after accounting for covariates in the context of other predictors, which was unexpected (Table 5, Appendix A). We assumed that education attainment was only marginally impacted by the COVID-19 pandemic, while workplace closures might have had a more profound impact on individuals’ SRMH. The results suggest the importance of considering multiple life aspects when assessing SRMH, as the pandemic, along with other major life circumstances, may have influenced these relationships in ways we could not anticipate.

In our study, we also found that women and younger participants had a worse self-assessment of mental health; however, it cannot be excluded that COVID-19-related distress also might have affected these results, because the COVID-19 pandemic produced adverse psychological consequences in the domain of mental health worldwide, especially among women and young people [26,29]. It was interesting to see that, in the sensitivity analysis, age had no statistically significant effect on SRMH for participants exposed to HD, in the context of other predictors that are obviously more important. We also found gender-specific consequences for SRMH, which are as follows: females exposed to HD reported an even worse mental health status than HD-exposed males, especially those who were negatively affected. This could mean that women were more likely to be subjected to mental health risks from a relative who drank excessively, which is a finding similar to other studies [2,19].

In the regression analysis of potentially risky predictors of SRMH related to drinking behaviors, the frequency of alcohol consumption in the past 12 months was found to be a significant predictor for those who did not live with an HD. Specifically, more frequent drinking was associated with worse SRMH (Table 4). Additionally, heavy episodic drinking (HED) was identified as a risky behavior that increased the likelihood of poor SRMH for individuals who did not live with an HD during childhood, and for those who did but were negatively affected. Furthermore, among those who lived with an HD, the risk of lower SRMH was attributed to individual drinking harms rather than drinking habits (Table 4). However, in sensitivity analyses (Table 5), we identified differences in SRMH prediction based on drinking habits, possibly reflecting different current drinking habits, between those who were negatively affected and those who were not, which may be influenced by adverse childhood experiences, as described in the literature [2]. This points to a more complex relationship between drinking habits and SRMH in individuals who lived with an HD. In general, finding a different form of association indicated that living with an HD during childhood is experienced in various ways and can be risky for SRMH, not only for those who were negatively affected, but also for those who reported no negative effects, if they engaged even in non-risky drinking habits.

The study found that childhood experiences of living with an HD can lead to different long-term effects and different predictors for SRMH later in life, depending on whether individuals perceived themselves as being negatively affected by a relative’s excessive drinking or not. The different perceptions between participants with opposing experiences is possibly due to interactional individual characteristics and their relationships, but also possibly due to a variety of social factors that require further consideration and research.

Within this line of work, scholars have demonstrated that different combinations of indicators increase the risk of poor mental health outcomes. Previous studies have also shown that not all children exposed to relatives’ alcohol consumption experienced negative effects, due to protective factors like older siblings, extended family, and school structure. Furthermore, some are more vulnerable than others to the difficult situations in the family environment, while others can develop resilience to cope with difficult situations that follow in their adult years [2,21,23].

Without clear evidence about the effects of others’ drinking at home, parents and health and education services may underestimate its harmful impacts on children [11]. Baldwin’s study highlights the importance of preventing childhood maltreatment to prevent psychopathology, but also points to other contributing factors [14,25]. Identifying subgroups at risk and understanding the complex interplay between ACEs and the moderating effects of family and community support can lead to targeted interventions [23].

The pathways between adverse childhood experiences and adverse health outcomes are not deterministic, and many individuals find a source of resilience in overcoming such adversities. However, the occurrence of ACEs did not necessarily predict problematic outcomes for all victims, especially if they experienced safe, stable, and nurturing relationships in their family or communities. Thus, protective factors can reduce or even offset the consequences of ACEs [12].

The descriptive analysis from this regional study revealed that the experience of living with a heavy drinker during childhood/teen years is not rare. A high prevalence was found, with negative effects, especially in Hungary (34.4%) and Croatia (26.5%), while the lowest prevalence was noted in Italy (7.8%); thus, there is a large variation among the observed countries, which is already documented in other European countries [12,23,30]. Understanding the prevalence of such childhood experiences and risk factors for mental health is a critical element in the comprehensive approach to prevention.

The results emphasize the need for early prevention and targeted interventions to reduce alcohol-related harm, especially among vulnerable groups. They highlight the importance of addressing individual and environmental factors in school and family settings, with attention to gender differences. By using a group-centered and cross-cultural approach, the study revealed how childhood experiences shape self-rated mental health (SRMH) and identified key predictors. These findings underscore the multifaceted nature of mental health outcomes and the necessity of evidence-based interventions to mitigate the long-term effects of others’ drinking, and also inform practices and policies for improved mental health outcomes.

While additional research may be needed to support causality, the authors suggest caution regarding heavy alcohol use within the household with children or teens, as there is a lasting effect on their mental health status. Longitudinal studies are needed to accurately assess and quantify the predictive influence of alcohol use among household members on the full scope of childhood well-being outcomes over time, including the enduring effects on mental health.

Several limitations to this study must be considered. Firstly, due to the nature of cross-sectional data, conclusions regarding causality cannot be drawn. The assessment of alcohol use by others during CH is based on self-reports and measured retrospectively; hence, it may have potentially been misrecalled or affected by social desirability biases. Typically, survey methods in estimating alcohol consumption are associated with underreporting [1], which could imply that consumption levels might be underestimated. Although SRMH is a useful tool for estimation of the mental health needs of populations, poor SRMH may not have universal meanings across ethnically diverse populations. Ethnic groups differ in how their poor SRMH reflects psychiatric conditions, and these ethnic differences may be a source of measurement bias in cross-ethnic health comparisons [31]. Another limitation is the lack of measures of parenting behaviors or other risk or protective factors affecting children. Furthermore, exposure cannot definitively be anchored to a specific time-point in childhood, although the survey referred to events occurring before age 18, so the assessment of exposure was not comprehensive and might underestimate the burden of childhood adversity in this sample. Lastly, the study cannot discuss an individual’s cumulative mental health history.

## 5. Conclusions

Given the link between childhood experiences with adult health and well-being, this study examined how living with a heavy drinker (HD) during childhood and/or adolescence affected self-rated mental health (SRMH) in adulthood. Assuming that exposure to an HD could have long-term effects on SRMH, the analysis examined differences in SRMH between individuals exposed to an HD and those who were not, as well as between those who were negatively affected and those who were not, in order to identify specific risk and protective factors for SRMH. Additionally, it explored the prevalence of living with an HD within a regional context and assessed the extent of its negative effects, considering cross-country differences.

The results revealed that the experience of living with a heavy drinker (HD), or someone who drank a lot heavily during childhood and/or teen years, was common in the adult general population, with a high prevalence of negative effects varying across countries.

Most adults who were exposed to an HD in the home environment during childhood perceived themselves as negatively affected and reported worse SRMH compared to those who did not live with a heavy drinker, highlighting an important public health concern. This experience was consistently associated with an increased risk of poor SRMH in adulthood, across all observed countries, demonstrating its persistent and lasting negative effects. Exposed individuals who were negatively affected reported poorer SRMH in the total sample compared to those who were not negatively affected, but analyses conducted separately by country suggested potential cultural differences and emphasized the need for further investigation.

The results identified relationship satisfaction (RAS) and self-rated general health (SRH) as the most significant protective factors contributing to better SRMH. Self-perceived relationship satisfaction (RAS) emerged as the strongest predictor of SRMH, particularly among individuals not negatively affected, and likely has the potential to buffer the negative effects of SRMH, underscoring the importance and benefits of good social connections. The results also suggested that women may be more vulnerable to mental health risks due to a relative’s excessive drinking.

Heavy episodic drinking (HED) was identified as a risky behavior that increased the likelihood of poor SRMH for both individuals who did not live with an HD during childhood and those who did, but were negatively affected. Additionally, more frequent drinking was associated with poorer SRMH among individuals who did not live with an HD. Differences in SRMH prediction based on drinking habits may have reflected varying current drinking habits between groups with different experiences of living with an HD, highlighting the complex relationships influencing SRMH in adulthood and emphasizing the need for future research.

Differences in SRMH prediction based on drinking habits may have reflected variations in current drinking behaviors between groups with different experiences of living with a heavy drinker (HD), highlighting the complex relationships influencing SRMH in adulthood and emphasizing the need for future research.

While children have little control over their environment, targeted public health and preventive measures are essential to protect those living with heavy drinkers. To mitigate the negative consequences of a home drinking environment, comprehensive mental health assessments and evidence-based interventions should be prioritized. Public health efforts that build resilience by providing adults and adolescents (and their parents) with tools, training, and support, while encouraging positive personal relationships, particularly within schools and families, are crucial for reducing alcohol-related harms and improving health and well-being.

## Figures and Tables

**Table 1:** Sociodemographic characteristics, as well as alcohol and health-behavioral variables of six countries (Austria, Bosnia and Herzegovina, Croatia, Hungary, Italy, Slovenia). N = 11,099.

			Austria	Bosnia and Herzegovina	Croatia	Hungary	Italy	Slovenia
	Variables	Category	n (%) ^1^	n (%) ^1^	n (%) ^1^	n (%) ^1^	n(%) ^1^	n (%) ^1^
Personal variables	Gender	Male	1515 (48.9)	719 (47.9)	720 (47.9)	958 (47.7)	748 (49.7)	745 (49.6)
		Female	1574 (50.9)	781 (52.1)	780 (51.9)	1047 (52.1)	755 (50.3)	757 (50.4)
	Age group	Younger (18–34)	1060 (34.2)	520 (34.7)	505 (33.6)	667 (33.2)	434 (28.9)	472 (31.4)
		Medium (35–49)	1109 (35.8)	543 (36.2)	562 (37.4)	810 (40.3)	642 (42.7)	606 (40.3)
		Older (50–64)	926 (29.9)	437 (29.1)	435 (29.0)	532 (26.5)	428 (28.5)	425 (28.3)
Socio-demographic variables	Education level	Low	31 (1.0)	279 (18.6)	19 (1.3)	95 (4.7)	31 (2.0)	62 (4.2)
		Medium	2326 (75.2)	969 (64.6)	838 (55.8)	1209 (60.2)	695 (46.2)	983 (65.4)
		High	738 (23.8)	252 (16.8)	645 (42.9)	705 (35.1)	778 (51.7)	458 (30.5)
	Professional activity	Non-active	925 (29.9)	814 (54.3)	490 (32.6)	588 (29.3)	542 (36.0)	424 (28.2)
		Active	2170 (70.1)	686 (45.7)	1012 (67.4)	1421 (70.7)	962 (64.0)	1079 (71.8)
	Household members	Living alone	838 (27.1)	344 (22.9)	160 (10.7)	342 (17.0)	206 (13.7)	215 (14.3)
		Living with someone	2257 (72.9)	1156 (77.1)	1342 (89.3)	1667 (83.0)	1298 (86.3)	1288 (85.7)
Living with an HD in childhood and teen years		Did not live with an HD	2287 (73.9)	1149 (76.6)	976 (65.0)	1193 (59.4)	1298 (86.3)	974 (64.8)
		Lived but not negatively affected	155 (5.0)	55 (3.7)	86 (8.5)	74 (3.7)	63 (4.2)	53 (3.5)
		Lived and negatively affected	571 (18.4)	286 (19.1)	398 (26.5)	692 (34.4)	117 (7.8)	428 (18.5)
Alcohol drinking behaviors	Frequency of alcohol drinking in the past 12 months	Never	244 (7.9)	513 (34.2)	73 (4.9)	133 (6.8)	57 (3.8)	141 (9.4)
		Rare	700 (22.6)	151 (10.1)	268 (17.9)	512 (25.5)	131 (8.8)	427 (28.4)
		Monthly	955 (30.9)	330 (22.0)	340 (22.6)	451 (22.5)	171 (11.4)	374 (24.9)
		Weekly	1062 (34.2)	358 (23.9)	717 (47.7)	828 (41.2)	913 (60.7)	507 (33.8)
		Daily	134 (4.3)	110 (7.3)	104 (6.9)	85 (4.2)	232 (15.4)	54 (3.6)
	Heavy episodic drinking in the last 12 months (HED)	Abstainers	244 (7.9)	513 (34.2)	73 (5.9)	133 (6.6)	57 (3.7)	141 (9.4)
		Never drank risky	848 (27.4)	697 (46.5)	262 (17.4)	597 (29.7)	422 (28.1)	496 (33.0)
		1+ time(s)	2003 (64.7)	287 (19.3)	1167 (76.7)	1279 (63.7)	1025 (68.2)	866 (57.6)
Personal drinking consequences	Individual harms for a drinker (RAPS)	Abstainers	244 (7.9)	513 (34.2)	73 (5.6)	133 (6.6)	57 (3.8)	141 (9.4)
		None (RAPS all negative)	2346 (75.8)	925 (61.7)	811 (61.7)	1371 (68.2)	1137 (75.6)	1138 (75.7)
		At least one (RAPS 1+)	505 (16.3)	62 (4.1)	430 (32.7)	505 (25.1)	310 (20.6)	224 (14.9)
Subjective well-being	Self-rated Satisfaction with relationships (RAS-1)	Poor to fair	545 (17.7)	84 (5.6)	329 (21.8)	801 (39.8)	384 (25.5)	315 (21.2)
		Good	2544 (82.2)	1414 (94.2)	1172 (78.2)	1206 (60.0)	1115 (74.2)	882 (78.6)
	Self-rated health (SRH)	Poor to fair	740 (24.0)	282 (18.9)	410 (27.3)	867 (43.2)	375 (24.9)	353 (23.4)
		Good	2349 (75.9)	1217 (81.1)	1089 (72.7)	1139 (56.7)	1124 (74.7)	1150 (76.5)
	Self-rated mental health (SRMH) *	Poor to fair	934 (30.1)	193 (12.9)	392 (26.1)	1111 (55.3)	564 (37.7)	365 (24.3)
		Good	2153 (69.5)	1307 (87.1)	1108 (73.9)	895 (44.6)	934 (62.1)	1138 (75.7)
Number of participants			3089	1500	1500	2005	1503	1502

^1^ regarding total number of respondents to the specific question; * variable of special interest.

**Table 2:** Mean difference between groups in self-rated mental health (SRMH) by childhood/teenage experience of living with a heavy drinker (CH1), and being negatively affected by their drinking (CH2), for all countries.

		N	M (SD)	t (df)	*p*	Mean Difference (CI)	Cohen’s d
CH1	No Yes	7877 3009	2.14 (0.909) 2.35 (0.980)	−10.3 (5102)	<0.001	−0.221 [−0.250–0.172]	0.227
CH2	Not negatively affected Negatively Affected	486 2492	2.17 (0.942) 2.39 (0.982)	−4.60 (706)	<0.001	−0.216 [−0.311–0.122]	0.222

CH1—During your childhood or teenage years, did you live with any person whom you consider to be a fairly heavy drinker or someone who drank a lot? No; Yes; CH2—How much were you negatively affected by this person/these persons’ drinking? Not negatively affected; Negatively Affected; SRMH—How would you rate your psychological well-being (SRMH)?

**Table 3:** Spearman Rho correlation coefficient between childhood and/or teenage experience of living with a heavy drinker and SRMH for the overall sample and for each country.

	Austria	Bosnia and Herzegovina	Croatia	Hungary	Italy	Slovenia	All Countries Together
CH1-SRMH correlation	0.068 **	0.086 **	0.108 **	0.092 **	0.077 **	0.105 **	0.100 ** N = 10,886
CH2-SRMH correlation	0.094 *	−0.045	0.136 **	0.037	−0.033	0.124 **	0.080 ** N = 2978

CH1—During your childhood or teenage years, did you live with any person whom you consider to be a fairly heavy drinker or someone who drank a lot? No; Yes; CH2—How much were you negatively affected by this person/these persons’ drinking? Not affected; Affected; SRMH—How would you rate your psychological well-being (SRMH)? ** *p* < 0.01; * *p* < 0.05.

**Table 4:** Odds ratio for poor SRMH in the two samples of those who did not experience living with a heavy drinker (HD) during childhood/teen years and those who did.

		SRMH—Did Not Experience Living with a Heavy Drinker in Childhood/Teen Years (*N = 5262)*			SRMH—Experienced Living with a Heavy Drinker in Childhood/Teen Years (*N =* 1859)		
Predictor (Reference Category)	Category	OR	*p*	95% CI	OR	*p*	95% CI
Gender (reference category: Male)	Female	1.285	<0.001	1.132–1.459	1.548	<0.001	1.264–1.897
Age group (reference category: 18–34)	35–49	0.857	0.461	0.568–1.292	0.878	0.289	0.690–1.117
	50–64	0.460	0.002	0.280–0.755	0.728	0.018	0.561–0.946
Professional activity (reference category: Non-active)	Active	0.738	<0.001	0.644–0.845	0.642	<0.001	0.517–0.798
People living in the household (reference category: Living alone)	Living with at least one person	0.853	0.052	0.727–1.001	-	-	-
Self-rated health (SRH) (reference category Good health)	Poor health	7.875	<0.001	5.484–11.310	5.242	<0.001	3.234–8.495
Self-rated Satisfaction with relationships (RAS-1) (reference category: Good relationships with others)	Poor relationships with others	13.300	<0.001	7.958–21.988	19.647	<0.001	8.463–45.614
Frequency of alcohol drinking in the past 12 months (reference category: Rarely)	Monthly	1.223	0.012	1.046–1.431	-	-	-
	Weekly	1.279	0.006	1.074–1.524	-	-	-
	Every day	1.795	0.033	1.047–3.076			
Heavy episodic drinking in the last 12 months (HED) (reference category: Abstainers)	Non-risky	1.437	<0.001	1.173–1.760	-	-	-
	1+ time(s)	1.811	<0.001	1.494–2.194	-	-	-
Individual harms for a drinker (RAPS) (reference category: 0)	1+	1.360	<0.001	1.254–1.475	1.207	<0.001	1.101–1.324
Nagelkerke R square		0.131			0.177		

Gender: 1: male; 2: female; age: 1: 18–34; 2: 35–49; 3: 50+; professional activity: 1: non-active; 2: active; people living in household: 1: living alone; 2: living with at least one person; self-rated general health: 1: good health; 2: poor health; relationships with others: 1: good relationships with other; 2: poor relationships with others; individual harms for a drinker RAPS: 0: no RAPS, 1+: one or more individual harms for a drinker; frequency of alcohol drinking in the past 12 months: 1: never; 2: rarely; 3: monthly; 4: weekly; 5: daily; heavy episodic drinking in the last 12 months, HED: 1: abstainers; 2: non-risky drinking; 3: 1+ time(s).

**Table 5:** Odds ratio for poor SRMH in the subsamples of those who experienced living with a heavy drinker (HD) during childhood/teen years and were not negatively affected and those who were.

SRMH of Those Who Experienced Living with a Heavy Drinker in Childhood/Teen Years							
		Not Negatively Affected (*N =* 332)			Negatively Affected (*N =* 1511)		
Predictor (Reference Category)	Category	OR	*p*	95% CI	OR	*p*	95% CI
Gender (reference category: Male)	Female	**-**	-	-	1.598	<0.001	1.273–2.006
Level of education (reference category: Low education)	Medium education	0.907	0.831	0.369–2.228	-	-	-
	High education	0.510	0.174	0.193–1.347	-	-	-
Professional activity (reference category: Non-active)	Active	0.747	0.285	0.437–1.275	0.590	<0.001	0.463–0.752
Self-rated health (SRH) (reference category: Good health)	Poor health	3.256	0.014	1.264–8.387	5.612	<0.001	3.156–9.978
Self-rated Satisfaction with relationships (RAS-1) (reference category: Good relationships with other)	Poor relationships with other	28.724	0.002	3.450–239.173	18.765	<0.001	7.433–47.372
Heavy episodic drinking in the last 12 months (HED) (reference category: Abstainers)	Non-risky	2.479	0.047	1.014–6.064	1.128	0.433	0.835–1.523
	1+ time(s)	2.083	0.096	0.878–4.943	1.346	0.037	1.017–1.782
Individual harms for a drinker (RAPS) (reference category: 0)	1+	1.231	0.111	0.954–1.590	1.197	<0.001	1.085–1.322
Nagelkerke R square		0.201			0.191		

Gender: 1: male; 2: female; education: 1: low; 2: medium; 3: high; professional activity: 1: non-active; 2: active self-rated general health: 1: good health; 2: poor health; relationships with others: 1: good relationships with other; 2: poor relationships with others; individual harms for a drinker, RAPS: 0: no individual harms for a drinker; 1+: one or more individual harms for a drinker; heavy episodic drinking in the last 12 months, HED: 1: abstainers; 2: non-risky drinking; 3: 1+ time(s).

## Data Availability

The owner of the SEAS-2 data is the Health and Digital Executive Agency (HaDEA) of the European Commission. The DEEP SEAS group, as contractors of the tendered service contract number 20177113, are authorized to share these data publicly, in line with HaDEA’s policy to provide open access to documents and data created with EU funding. Researchers interested in using these data can receive the public file from the DEEP SEAS website (https://www.deep-seas.eu/standard-eu-alcohol-survey/ accessed on 16 May 2023), and should request permission to use the data, before any publication, via the following email: hadea-hp-tender@ec.europa.eu.

## Appendix A

**Table A1:** Spearman correlation between predictor variables and self-rated mental health (SRMH) according to whether participants did/did not live with fairly heavy drinkers/someone who drank a lot.

CH1		Gender	Age	Education	Professional Activity	People Living in the Household	Self-Rated General Health	Self-Rated Satisfaction with Relationships	Frequency of Alcohol Drinking in the Past 12 Months	Heavy Episodic Drinking in the Last 12 Months (HED)	Individual Harms for a Drinker (RAPS)
No	SRMH	0.067 **	0.030 **	−0.22	−0.103 **	−0.028 *	0.522 **	0.479 **	0.023 *	0.070 **	0.125 **
Yes	SRMH	0.116 **	0.033 **	−0.23	−0.135 **	0.020	0.506 **	0.485 **	−0.14	0.044	0.140 **

CH1—During your childhood or teenage years, did you live with any person whom you consider to be a fairly heavy drinker or someone who drank a lot? SRMH—How would you rate your psychological well-being? * *p* < 0.05; ** *p* < 0.01.

**Table A2:** Spearman correlation between predictor variables and self-rated mental health (SRMH) according to whether participants were/were not negatively affected by another persons’ drinking.

CH2		Gender	Age	Education	Professional Activity	People Living in the Household	Self-Rated General Health	Self-Rated Satisfaction with Relationships	Frequency of Alcohol Drinking in the Past 12 Months	Heavy Episodic Drinking in the Last 12 Months (HED)	Individual Harms for a Drinker (RAPS)
No	SRMH	0.079	−0.048	−0.152 **	−0.114 *	0.010	0.491 **	0.417 **	0.031	0.144 *	0.140 *
Yes	SRMH	0.115 **	−0.018	−0.008	−0.132 **	0.020	0.510 **	0.490 **	−0.023	0.062 *	0.130 **

CH2—How much were you negatively affected by this person/these persons’ drinking? Were you affected a lot, a little or not affected at all? SRMH—How would you rate your psychological well-being; * *p* < 0.05; ** *p* < 0.01.
